# Supplementary material for: Non-compulsory measures sufficiently reduced human mobility in Tokyo during the COVID-19 epidemic
Source: Sci Rep. 2020 Oct 22;10:18053. doi: 10.1038/s41598-020-75033-5 (PMC7581808; doi:10.1038/s41598-020-75033-5)
Supplement: Supplementary file 1 — Supplementary Information. [file 41598_2020_75033_MOESM1_ESM.pdf]

Supplementary Material

Non-Compulsory Measures Sufficiently Reduced Human Mobility in Tokyo during the COVID-19 Epidemic

Takahiro Yabe, Kota Tsubouchi, Naoya Fujiwara, Takayuki Wada, Yoshihide Sekimoto, Satish Ukkusuri*

*sukkusur@purdue.edu

This Supplementary Material contains the following:

- Supplementary Tables S1 - S2
- Supplementary Figures S1 - S8

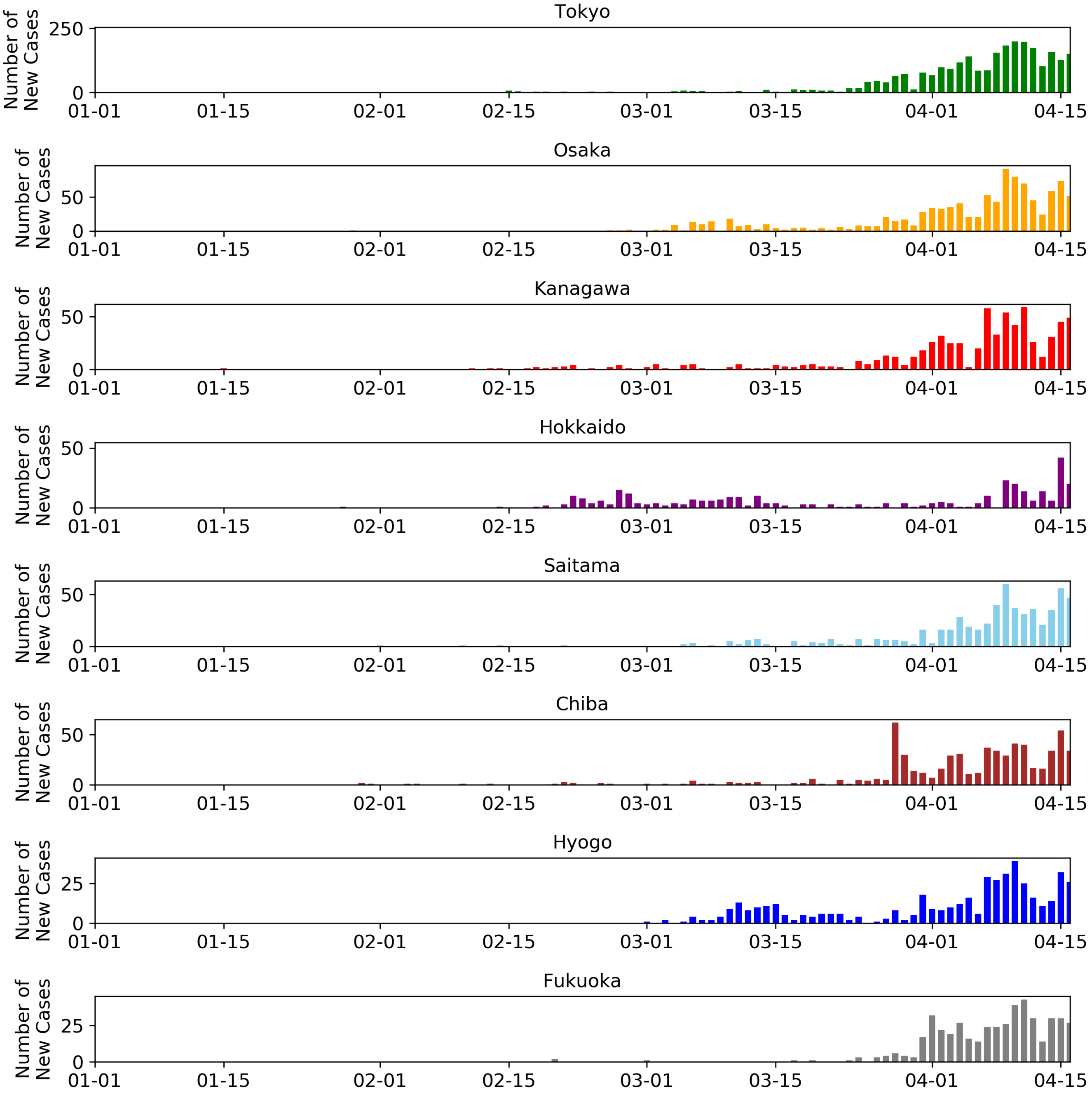

Figure S1. Number of new COVID-19 cases in major cities in Japan. For many of the cities in Japan, COVID-19 patients started to increase in the beginning of April.

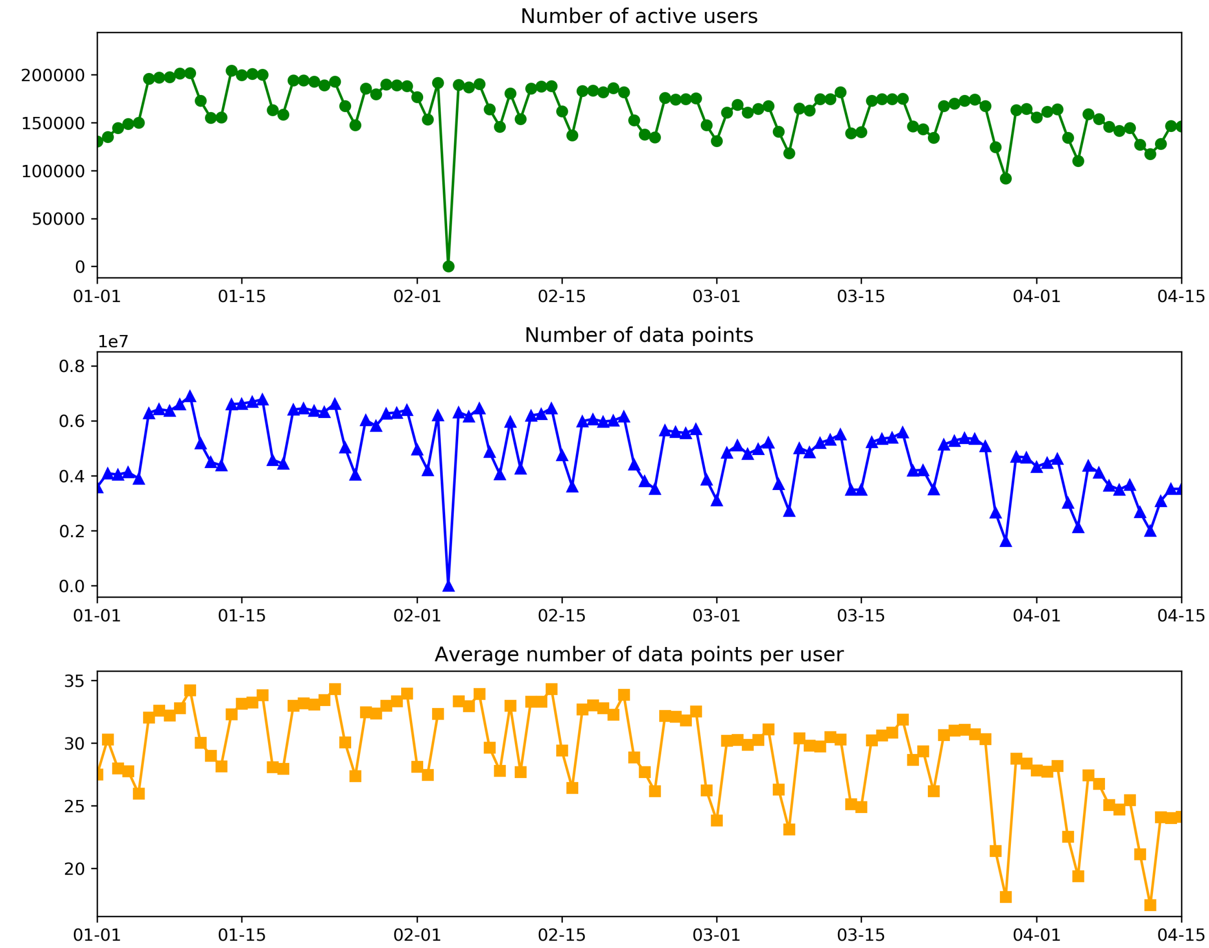

Figure S2. **Number of active IDs and total GPS observation points.** (Top) The number of active users slightly decrease as time passes, however stays stable for the time period. (B) Total number of GPS observations in the dataset. (C) Average number of data points per user decreases towards the end of March and in April since more users spend more time staying static.

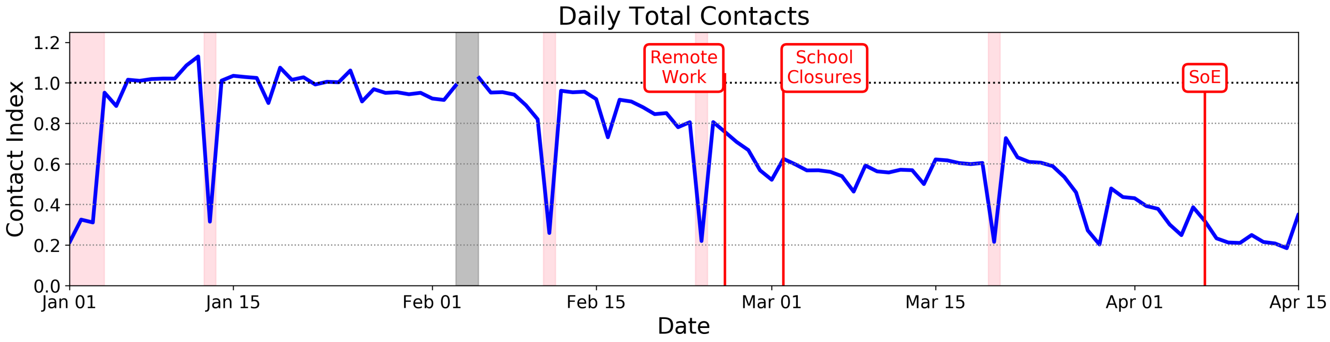

Figure S3. Social contact index normalized with respect to day of week. Averages of usual social contact index were computed by taking the average values on each day except January 13th, which was a holiday in Japan.

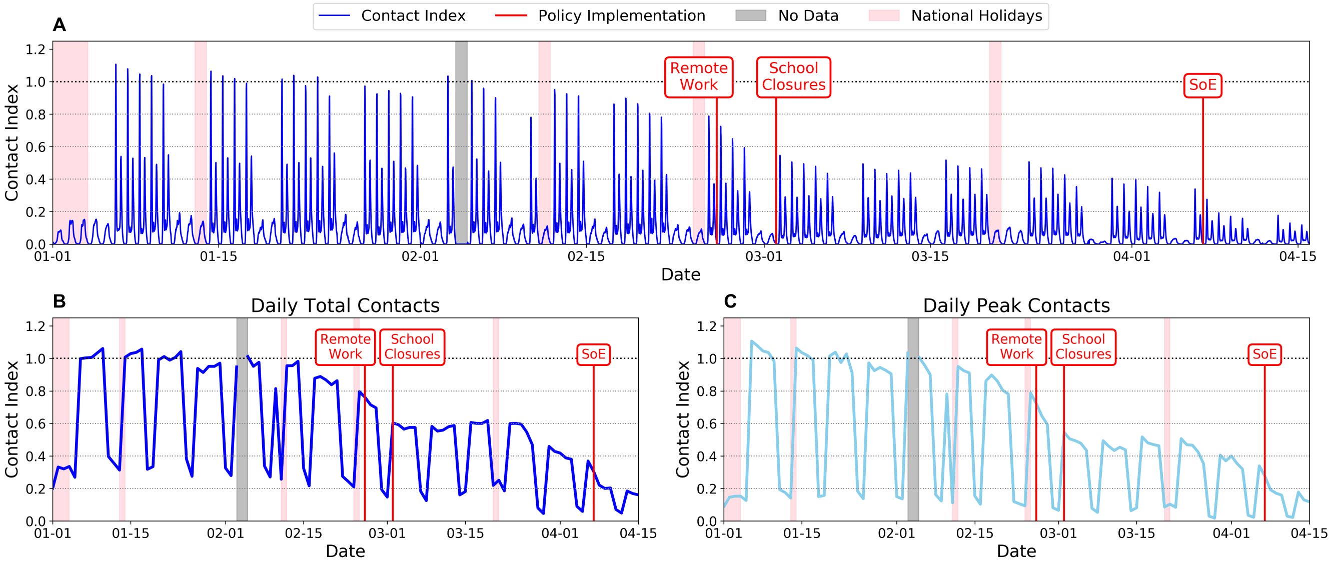

Figure S4. Social contact index with sptial threshold of 250 meters. (A) social contact index computed with the same method as Figure 3 (in main text), but with 250 meters as the spatial threshold. (B) and (C) show daily total and peak contacts. Regardless of the spatial threshold, the conclusion that more than 80% of the contacts were reduced stays consistent.

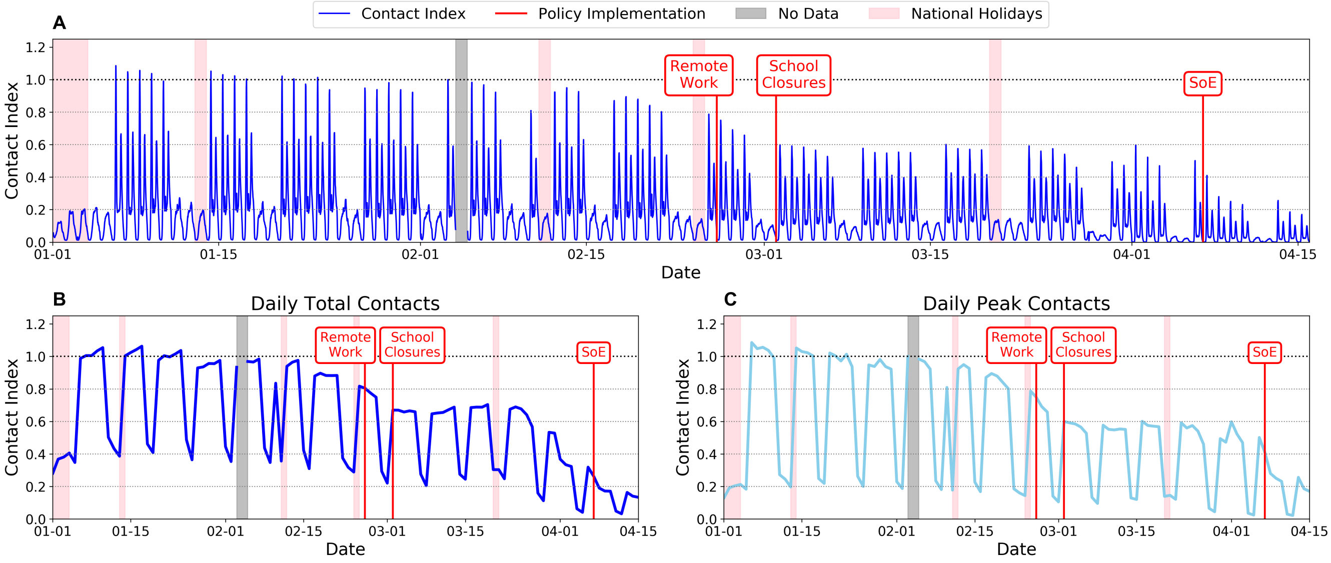

(a) Spatial threshold of 250 meters

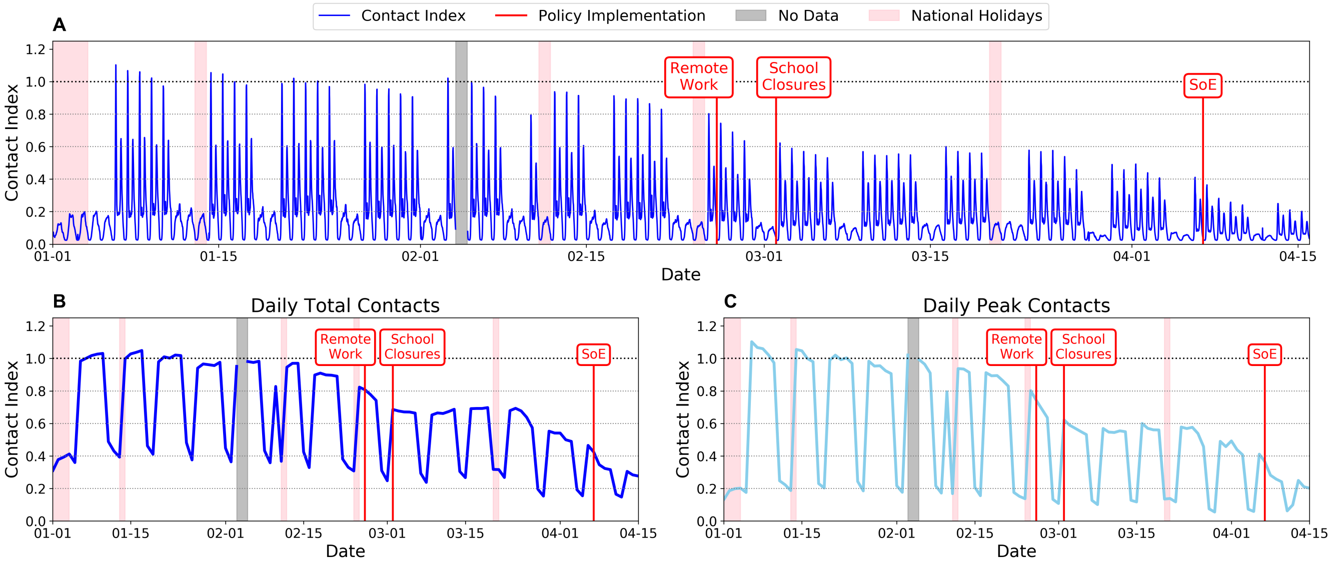

(b) Spatial threshold of 100 meters

Figure S5. Social contact index when including the users who are staying home.

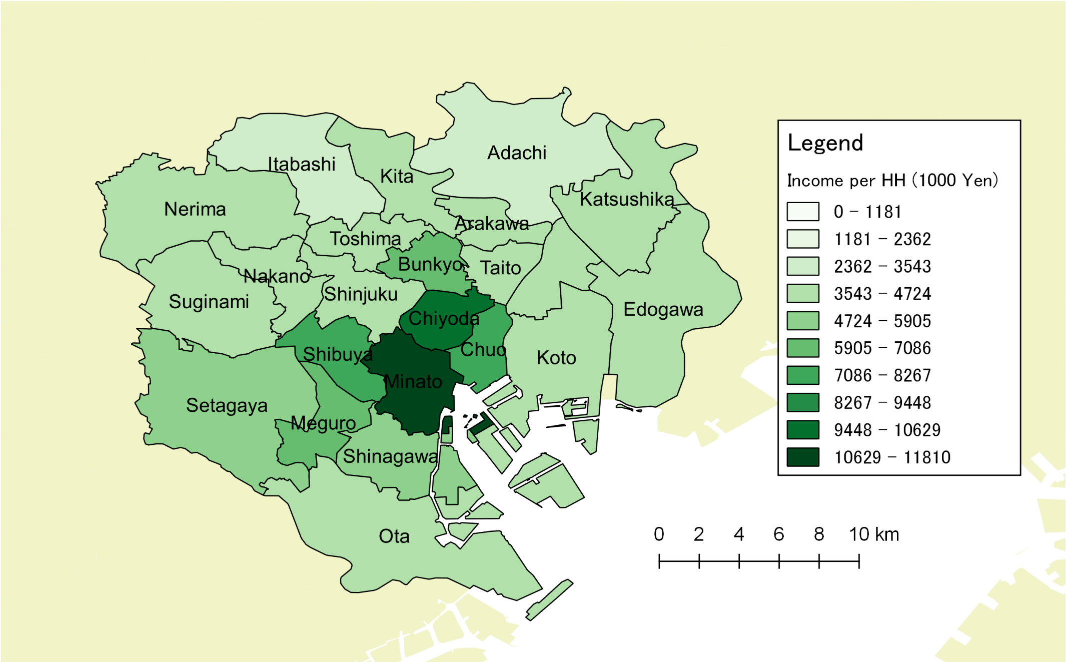

Figure S6. Spatial location of 23 wards in central Tokyo, and average household taxable income.

Table S1. Statistics showing the number of households and taxable income per household in all 23 wards in Tokyo.

Name	code	Total Taxable Income	Households	Mean Taxable Income (Thousand Yen/household)
Chiyoda	13101	333664476	33262	10031.4
Chuo	13102	562306834	79272	7093.3
Minato	13103	1541982291	130562	11810.3
Shinjuku	13104	926402861	204989	4519.2
Bunkyo	13105	718111843	120858	5941.7
Taito	13106	433617929	112117	3867.5
Sumida	13107	526774130	130862	4025.4
Koto	13108	1123594742	243708	4610.4
Shinagawa	13109	1011051510	212374	4760.7
Meguro	13110	945905092	146162	6471.6
Ota	13111	1648342738	371149	4441.1
Setagaya	13112	2620976315	463632	5653.1
Shibuya	13113	1029439108	135749	7583.4
Nakano	13114	748424622	196132	3815.9
Suginami	13115	1443707240	312001	4627.2
Toshima	13116	663702757	176376	3762.9
Kita	13117	658834551	178379	3693.4
Arakawa	13118	381267820	103101	3698.0
Itabashi	13119	1030999559	291408	3537.9
Nerima	13120	1505429630	337987	4454.1
Adachi	13121	1092439739	310662	3516.4
Katsushika	13122	761907030	201380	3783.4
Edogawa	13123	1202944874	309072	3892.1

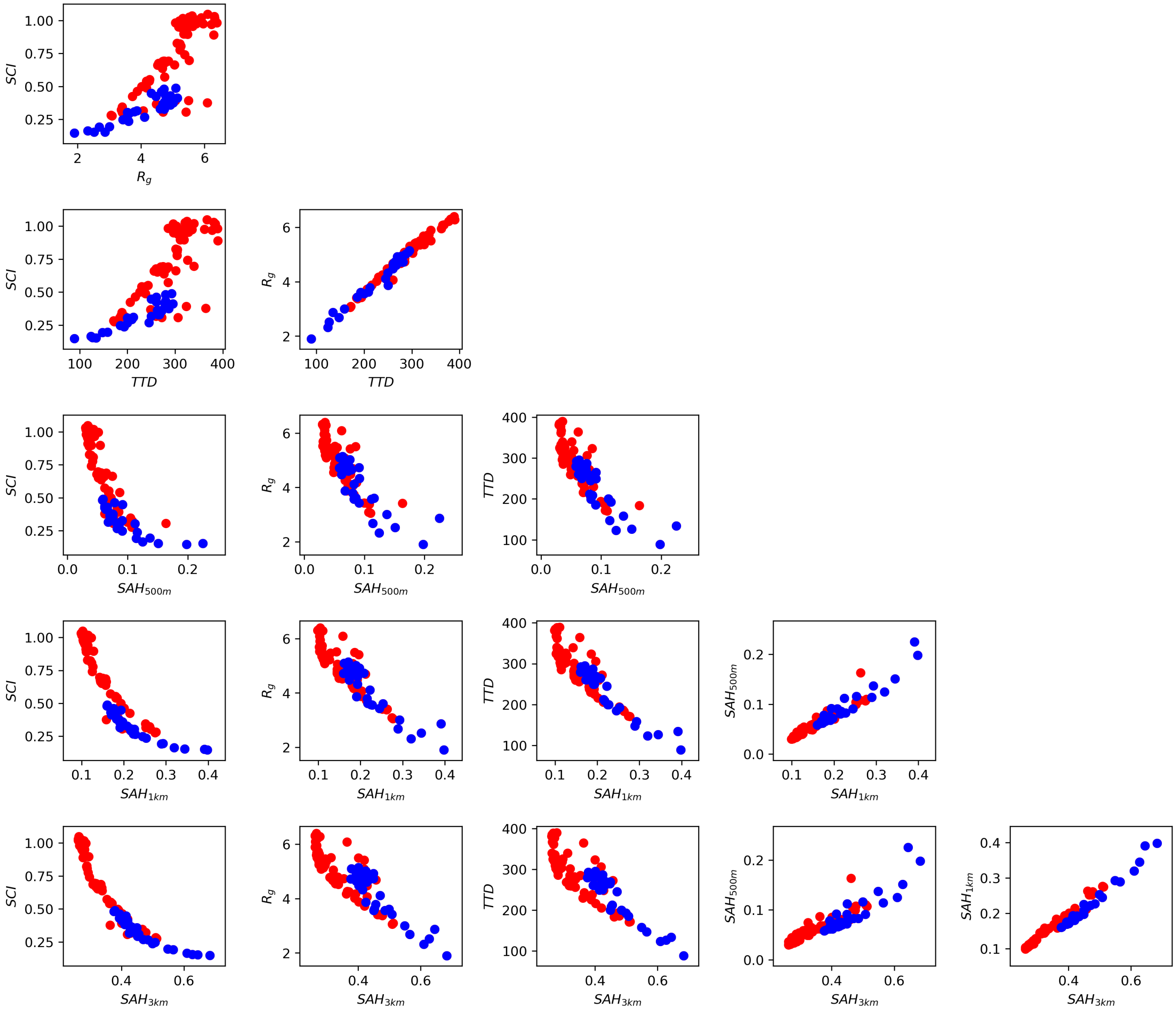

Figure S7. **Correlation between mobility metrics.** Red and blue scatter plots show the data points on weekdays and weekends, respectively.

Table S2. Location information of place of interests used in the analysis for Figure 4 and their types.

Type	Name	Location (longitude, latitude)	Main meshcode
Business districts	Tokyo Midtown	(139.73081589,35.66608288)	5339-3598-3
	Roppongi Hills	(139.72995758,35.66036471)	5339-3598-1
	East Shinagawa	(139.73868833,35.62842595)	5339-3559-1
	West Shinjuku	(139.69789982,35.68982286)	5339-4525-4
	Toranomon Hills	(139.74918365,35.66695452)	5339-4509-2
	Kioicho	(139.73716736, 35.67953989)	5339-4518-4
Shopping areas	Ginza	(139.76255138,35.67158177)	5339-4601-3
	Omotesando	(139.71174419,35.66523219)	5339-3596-4
	Shibuya Center Street	(139.69501934,35.65879283)	5339-3595-2
	Shinjuku Golden Street	(139.70463753,35.69393568)	5339-4536-1
	Akihabara Street	(139.7715995,35.7007106)	5339-4641-2
	Kagurazaka	(139.73385577,35.70388258)	5339-4548-2
Parks	Shinjuku Gyoen	(139.71004486,35.68518698)	5339-4526-2
	Yoyogi Park	(139.69665527,35.67159146)	5339-4505-4
	Hamarikyu	(139.76347446,35.65975451)	5339-3691-1
	Ueno Park	(139.77398872,35.71550708)	5339-4651-4
	Shiba Park	(139.74798203,35.65492509)	5339-3589-4
Stations	Shinjuku	(139.7008754,35.68957156)	5339-4526-3
	Shibuya	(139.70186152,35.65800855)	5339-3586-3
	Shinagawa	(139.73868833,35.62842595)	5339-3559-1
	Tokyo	(139.76641937,35.6812414)	5339-4611-3
	Akihabara	(139.77305991,35.6986063)	5339-4631-4
Airport	Haneda Airport	(139.77973938,35.54954667)	5339-2652-3

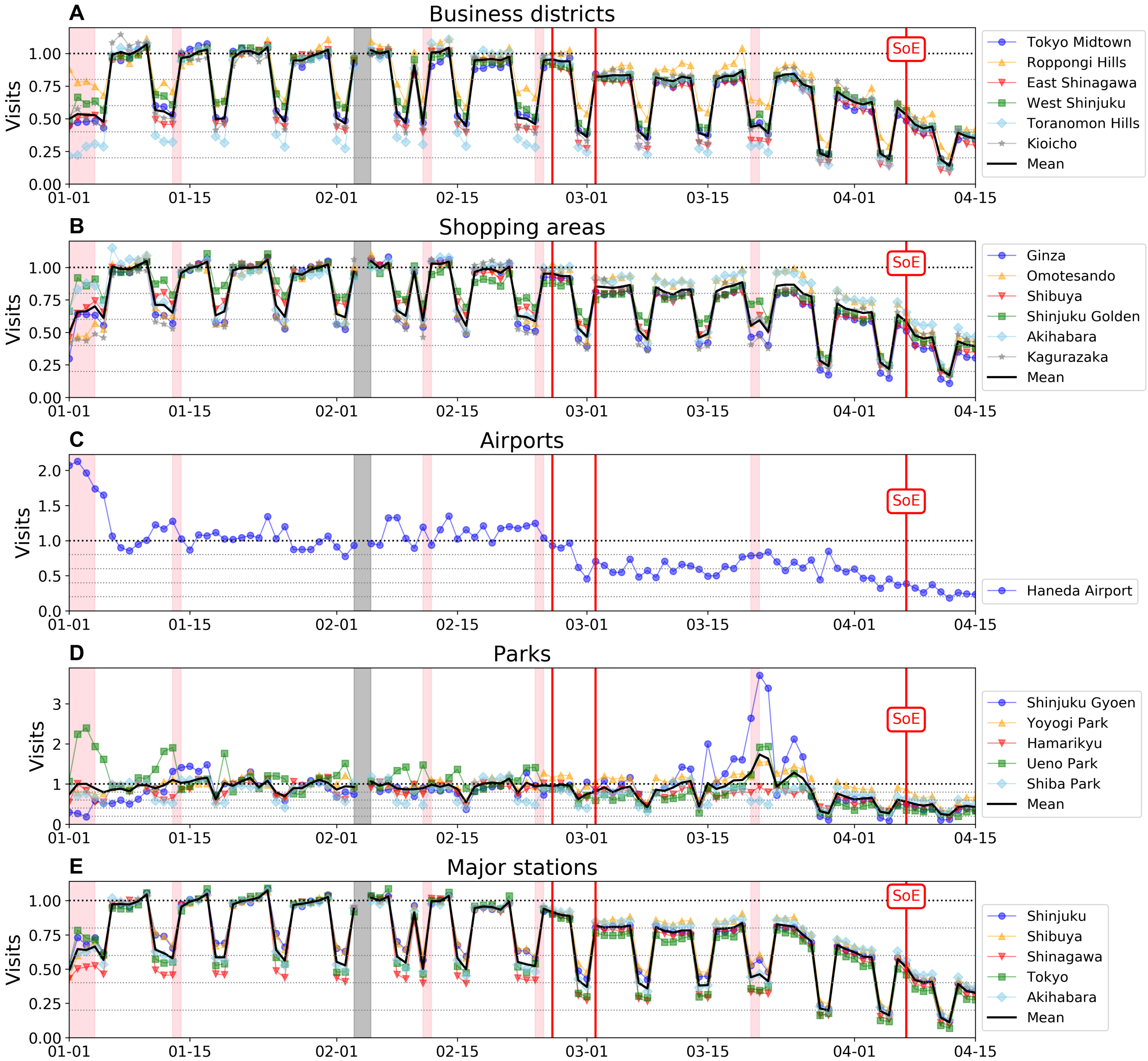

Figure S8. **Relative amount of visits to popular point-of-interests in Tokyo.** Amount of visits normalized by the weekdays in January for popular POIs are shown. (A) business districts, (B) shopping areas, (C) Haneda airport, (D) major parks, and (E) major stations.
